# Portal venous blood flow velocity is a factor associated with portal venous thrombosis after partial splenic artery embolization in hepatic cirrhosis patients

**DOI:** 10.1097/MS9.0000000000001577

**Published:** 2023-12-11

**Authors:** Jiaming Huang, Haifeng Liu

**Affiliations:** a Department of Gastroenterology, Ganzhou People’s Hospital; b Department of Gastroenterology, Xinfeng People’s Hospital, Ganzhou, Jiangxi, China

**Keywords:** hepatic cirrhosis, hypersplenism, partial splenic artery embolization, portal venous blood flow velocity, portal venous thrombosis

## Abstract

**Objective:**

To investigate risk factors for portal venous thrombosis (PVT) after partial splenic artery embolization (PSE) in hepatic cirrhosis patients.

**Methods:**

The authors retrospectively analyzed 151 hepatic cirrhosis patients with hypersplenism who underwent partial splenic artery embolization between January 2020 and December 2021. The patients were divided into a PVT group and a non-PVT group according to whether they had PVT after PSE. Univariate analyses were performed to select risk factors for PVT after PSE, and multivariate analysis was used to analyze variates with a value of *P* less than 0.1 in univariate analysis.

**Results:**

There were 151 patients enroled in the study, with 22 patients in the PVT group and 129 patients in the non-PVT group. There was no significant difference in terms of age, sex, smoking, hypertension, diabetes, Child–Pugh between two groups. White blood cell (WBC) and platelet counts after PSE were significantly higher than those before PSE in both the PVT group and non-PVT group. Univariate analysis showed that portal venous blood flow velocity, ligation of oesophageal varices and WBC after PSE were found to have a *P* value less than 0.1. Multivariate analysis showed that portal venous blood flow velocity was a factor associated with PVT after PSE.

**Conclusion:**

Portal venous blood flow velocity was a factor associated with PVT after PSE. Portal venous blood flow velocity should be considered before patients undergo PSE.

## Introduction

Highlights

- The study investigated risk factors for portal venous thrombosis (PVT) after partial splenic artery embolization (PSE) in hepatic cirrhosis patients.
- Multivariate analysis showed that portal venous blood flow velocity was a factor associated with PVT after PSE.
- Portal venous blood flow velocity was a factor associated with PVT after PSE.
- Portal venous blood flow velocity should be considered before patients undergo PSE.

Hepatic cirrhosis is a widely prevalent disease with both high morbidity and mortality rates. There are many complications of hepatic cirrhosis, and complications are the leading cause of hepatic cirrhosis patient death^1–3^. Complications include hepatic encephalopathy, upper gastrointestinal bleeding, ascites, hepatorenal syndrome, spontaneous peritonitis, hepatopulmonary syndrome, portal venous thrombosis (PVT), etc. In addition, hepatic cirrhosis is often accompanied by hypersplenism, whose manifestations are decreased white blood cell (WBC) count, haemoglobin and platelet count.

Partial splenic artery embolization (PSE) is widely used as a treatment for hypersplenism in hepatic cirrhosis patients. Although effective in treating hypersplenism, PSE can cause complications such as PVT, splenic abscess, fever and pain^4–6^. Currently, there is few data about the prevalence of PVT following PSE in patients with hepatic cirrhosis. A study conducted by Ogawa *et al.* ^7^ found that 9 patients showed appearance or growth of thrombusin all 67 patients who underwent partial splenic artery embolization. The development of PVT is associated with an increased risk of death^8–10^. It is important to assess the risk factors for PVT after PSE in hepatic cirrhosis patients.

Studies on risk factors for PVT after PSE have mainly focused on the infarcted splenic volume and Child–Pugh score^4,6,11^. No studies have described portal venous blood flow velocity as a risk factor for PVT after PSE. The purpose of this study was to investigate the relationship between portal venous blood flow velocity and PVT after PSE in hepatic cirrhosis patients.

## Materials and methods

### Patients

This was a retrospective clinical study approved by the ethics committee of our hospital, the Institutional Review Board no. is TY-ZKY-021-01. The study was conducted in accordance with the Declaration of Helsinki. Informed consent was obtained from all patients. The study was registered. The clinical trial number was ChiCTR2100048291 (https://www.chictr.org.cn/bin/userProject). The work has been reported in line with the STROCSS criteria^12^.

**Figure 1:**
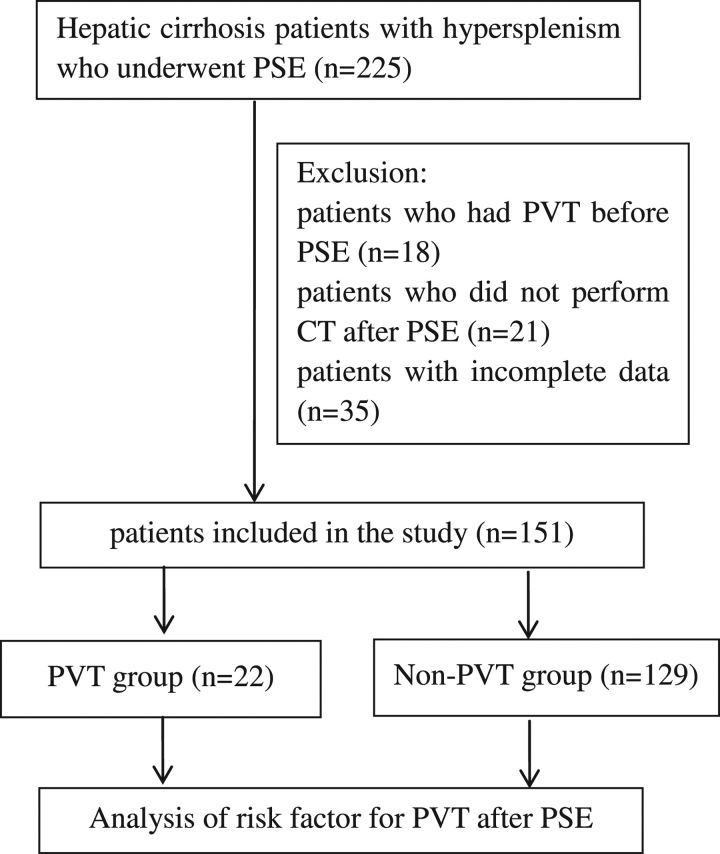
Flowchart of the study. CT, computed tomography; PSE, partial splenic artery embolization; PVT, portal venous thrombosis.

Data from hepatic cirrhosis patients with hypersplenism who underwent PSE between January 2017 and December 2021 were collected. The inclusion criteria were as follows: (a) patients diagnosed with hepatic cirrhosis with hypersplenism and (b) patients who received PSE treatment. Exclusion criteria were as follows: (a) patients who had PVT before PSE; (b) patients who did not perform computed tomography (CT) after PSE; and (c) patients with incomplete data. Patients were diagnosed with hepatic cirrhosis by abdominal ultrasound, CT or MRI. Hypersplenism was dignosed if patients had decrease of WBC, haemoglobin and platelet. The patients were divided into a PVT group and a non-PVT group according to whether they had PVT after PSE. Patients were followed up until June 2022 (Figure 1).

### PSE procedure

Under local anaesthesia, a 5.0-Fr sheath was inserted into the common femoral artery. The coeliac artery was selected using a 4.0-Fr catheter. A 2.2-Fr microcatheter was coaxially advanced into the splenic artery, and the tip of the catheter was inserted as distally as possible into the splenic artery. Embolization microspheres (500–700 µm, Hengrui Pharmaceutical) were injected slowly, and the embolized area was controlled between 30% and 50%, and was assessed by arteriography at the end of the procedure. Then, gentamicin (80 000 U, Qingfeng Pharmaceutical) and dexamethasone sodium phosphate (10 mg, Tianyao Pharmaceutical) were injected slowly to prevent infection and inflammation. After the procedure, patients were sent back to the ward to rest in bed for 8 hours.

### Evaluation items

Before treatment, the following data of all patients were obtained within 24 h after admission: previous history, smoking, drinking, white blood cell (WBC) count, haemoglobin, platelet count, total bilirubin, alanine aminotransferase (ALT), albumin, partial thromboplastin time (PT), activated partial thromboplastin time (APTT), fibrinogen, D-dimer, hepatic encephalopathy, ascites, urea nitrogen, creatinine, heart rate, systolic blood pressure, diastolic blood pressure, PVT, portal venous diameter, and portal venous blood flow velocity. Portal venous diameter, and portal venous blood flow velocity were calculated by Colour Ultrasound at a median of 16 days (range 1–65 days) before PSE. Blood samples were taken for examination of WBCs, haemoglobin and platelets 48–72 h after PSE. Contrast-enhanced CT was performed at a median of 18 days (range, 7–30 days ) to evaluate the presence of PVT after PSE.

### Statistical analysis

SPSS 26.0 software was used for analysis. Continuous variables are presented as the mean±standard deviation and were analyzed using Students *t*-test or rank sum test, as appropriate. Categorical variables are presented as frequencies and were assessed using the chi-square test. Univariate analyses were performed to select risk factors for PVT after PSE, and variates with a value of *P* less than 0.1 were used for multivariate analysis. *P* less than 0.05 was considered statistically significant.

## Results

### Baseline characteristics of patients

There were 151 patients included in the study; among them, 93 patients were male, mean age was 53 ± 10 years (range, 33–79 years). The causes of hepatic cirrhosis were hepatitis B virus infection in 97 patients (64.24%), alcohol consumption in 10 patients (6.62%), autoimmune hepatitis in 6 patients (3.97%), hepatitis B virus and alcohol consumption in 18 patients (11.92%), and unclassifiable or unknown in 20 patients (13.25%) (Table 1).

**Table 1:** Baseline characteristics of patients

Characteristics	Data (*n*=151)
Age (＞65), *n* (%)	23 (15.23%)
Sex (male), *n* (%)	93 (61.59)
AEtiology	
Hepatitis B virus, *n* (%)	97 (64.24)
Alcohol intake, *n* (%)	10 (6.62)
Autoimmune hepatitis, *n* (%)	6 (3.97)
Hepatitis B virus and alcohol consumption, *n* (%)	18 (11.92)
Unclassifiable or unknown aetiology, *n* (%)	20 (13.25)
Hypertension, *n* (%)	16 (10.60)
Diabetes, *n* (%)	23 (15.23)
Coronary heart disease	8 (5.30)
Stroke, *n* (%)	8 (5.30)
Chronic kidney disease, *n* (%)	3 (1.99)
Chronic obstructive pulmonary disease, *n* (%)	3 (1.99)
Hypothyroidism, *n* (%)	2 (1.32)
Peptic ulcer, *n* (%)	10 (6.62)
Ligation of oesophageal varices, *n* (%)	49 (32.45)
Smoking, *n* (%)	23 (15.23)
Child-Pugh (A/B/C)	84/58/9
Laboratory tests before PSE	
Total bilirubin (umol/l)	30.89±22.24
ALT (U/l)	27.29±14.45
Albumin (g/l)	34.43±5.51
Urea nitrogen (mmol/l)	6.01±7.16
Creatinine (umol/l)	92.42±136.27
PT (s)	14.82±1.95
Fibrinogen (g/l)	1.66±0.50
APTT (s)	35.88±32.73
dimer (mg/l)	1.66±4.17
Portal venous width (mm)	14.59±3.01
Portal venous blood flow velocity (cm/s)	16.72±4.52

ALT, alanine aminotransferase; APTT, activated partial thromboplastintime; PT, partial thromboplastin time.

### Comparison of WBCs, haemoglobin and platelets before and after PSE

WBC, haemoglobin and platelet counts before and after PSE were compared between the PVT group and non-PVT group. WBC counts after PSE were significantly higher than WBC counts before PSE in both the PVT group (*P*=0.005) and the non-PVT group (*P*=0.000). There were no significant differences between haemoglobin after PSE and haemoglobin before PSE in either the PVT group or the non-PVT group. Platelets after PSE were significantly higher than WBCs before PSE in both the PVT group (*P*=0.018) and the non-PVT group (*P*=0.000) (Table 2).

**Table 2:** Comparison of WBC, haemoglobin and platelet before and after PSE

Group	WBC before PSE (×109/l)	WBC after PSE (×109/l)	*P*
PVT group	2.63±2.11	5.75±2.23	0.005
Non-PVT group	2.67±1.25	7.89±3.31	0.000
	Haemoglobin before PSE (g/l)	Haemoglobin after PSE (g/l)	
PVT group	98.41±16.31	102.79±16.54	0.421
Non-PVT group	112.62±110.38	105.16±26.34	0.397
	Platelet before PSE (×109/l)	Platelet after PSE (×109/l)	
PVT group	40.43±12.14	64.26±21.56	0.018
non-PVT group	41.71±21.31	68.47±33.52	0.000

PSE, partial splenic artery embolization; PVT, portal venous thrombosis; WBC, white blood cell.

### Comparison of variates between the PVT group and the non-PVT group

There were more patients who had ligation of oesophageal varices in the PVT group than in the non-PVT group (*P*=0.010) . WBC after PSE (*P*=0.036) and portal venous blood flow velocity (*P*=0.001) were higher in the non-PVT group than in the PVT group. There was no significant difference between the PVT group and the non-PVT group in variates, such as, age, sex, smoking, hypertension, diabetes, Child–Pugh, WBC etc. (Table 3).

**Table 3:** Comparison of variates between PVT group and non-PVT group

Variates	PVT group (*n*=22)	non-PVT group (*n*=129)	*P*
Age (＞65 years), *n* (%)	2 (9.10)	21 (16.28)	0.386
Sex (male), *n* (%)	17 (77.27)	76 (58.91)	0.102
Smoking, *n* (%)	5 (22.73)	18 (13.95)	0.290
Hypertension, *n* (%)	1 (4.55.0)	15 (11.63)	0.318
Diabetes, *n* (%)	2 (9.09)	21 (16.28)	0.386
Ligation of oesophageal varices, *n* (%)	12 (54.55)	35 (27.13)	0.010
Child–Pugh, *n* (%)			0.480
A class	10 (45.45)	74 (57.36)	
B class	11(50.0)	47 (36.43)	
C class	1 (4.55)	8 (6.20)	
WBC before PSE (×109/L)	2.65±2.01	2.72±1.23	0.840
Haemoglobin before PSE (g/l)	97.47±18.69	112.42±110.71	0.615
Platelet before PSE (×109/l)	40.21±12.22	43.51±21.14	0.712
WBC after PSE (×109/l)	5.58±2.13	7.96±3.44	0.036
Haemoglobin after PSE (g/l)	112.70±19.44	105.16±22.28	0.785
Platelet after PSE (×109/l)	60.24±23.41	65.46±37.31	0.658
Total bilirubin (umol/l)	24.81±5.21	31.18±24.69	0.462
ALT (U/l)	27.62±10.52	27.29±14.05	0.894
Albumin (g/l)	34.67±5.04	34.12±5.11	0.772
Urea nitrogen (mmol/l)	5.25±1.42	6.33±7.71	0.736
Creatinine (umol/l)	74.92±11.61	96.46±140.25	0.627
PT (s)	14.62±1.12	14.84±2.16	0.844
Fibrinogen (mg/l)	1.51±0.35	1.67±0.51	0.363
APTT (s)	31.55±2.71	37.56±32.21	0.563
D-dimer (mg/l)	3.12±6.12	1.53±3.20	0.249
Portal venous width (mm)	15.14±1.70	14.52±3.21	0.413
Portal venous blood flow velocity (cm/s)	12.55±3.41	17.72±4.17	0.001

ALT, alanine aminotransferase; APTT, activated partial thromboplastin time; PSE, partial splenic artery embolization; PT, partial thromboplastin time; PVT, portal venous thrombosis; WBC, white blood cell.

### Univariate analysis of factors associated with PVT after PSE

Univariate analysis was performed to assess the risk factors. Portal venous blood flow velocity (*P*=0.004) was found to be associated with PVT after PSE, and patients with higher portal venous blood flow velocity were less likely to have PVT after PSE. Ligation of oesophageal varices (*P*=0.074) and WBC (*P*=0.065) after PSE were found to have a *P* value less than 0.1 (Table 4).

**Table 4:** Univariate analysis of risk factors for PVT after PSE

		Exp (B) 95% CI		
Variates	Exp (B)	Lower limit	Upper limit	*P*
Ligation of oesophageal varices	3.78	0.915	15.879	0.074
WBC after PSE	0.784	0.585	1.016	0.065
Portal venous blood flow velocity	0.696	0.535	0.897	0.004

Exp, experiment; PSE, partial splenic artery embolization; PVT, portal venous thrombosis; WBC, white blood cell.

### Multivariate analysis of factors associated with PVT after PSE

Risk factors with a *P* value less than 0.1 in univariate analysis were included in multivariate analysis. The results showed that portal venous blood flow velocity was associated with PVT after PSE (*P*=0.004) (Table 5).

**Table 5:** Multivariate analysis of risk factors for PVT after PSE

		Exp (B) 95% CI		
Variates	Exp (B)	Lower limit	Upper limit	*P*
Ligation of oesophageal varices	3.48	0.414	28.514	0.232
WBC after PSE	0.664	0.427	1.028	0.064
Portal venous blood flow velocity	0.567	0.377	0.854	0.005

Exp, experiment; PSE, partial splenic artery embolization; PVT, portal venous thrombosis; WBC, white blood cell.

## Discussion

PVT is a complication of not only hepatic cirrhosis but also PSE. Hepatic cirrhosis patients with PVT are at high risk of mortality^8,9^. As PSE is a frequently used treatment for hypersplenism in hepatic cirrhosis patients, it is of great significance to identify factors associated with PVT after PSE.

PSE and splenectomy are two main treatments for hypersplenism, but patients are more willing to accept PSE because PSE is an effective method with minimal trauma. In this study, PSE was successful in elevating patients’ WBC and platelet counts.

Several studies have been performed to assess risk factors for PVT after splenectomy. Preoperative splenic vein diameter is a risk factor for postsplenectomy PVT^13,14^. Kuroki speculated that stagnation of blood flow in the splenic vein may be associated with PVT after splenectomy^15^. In this study, results showed that portal venous blood flow velocity was a factor associated with PVT after PSE. Patients with higher portal venous blood flow velocity were less likely to have PVT after PSE, whereas patients with lower portal venous blood flow velocity were more likely to have PVT, which was the same as what Kuroki had speculated.

Several studies have reported that splenic infarction percentages and splenic infarction volume are associated with PVT^4,6,7,15^. Zhu *et al.* ^16^ reported that a percentage of infarcted spleen greater than 70% could significantly increase the likelihood of PVT. However, in this study, the percentage of infarcted spleen in all patients was controlled roughly within 30–50%, so the percentage of infarcted spleen was not assessed as a risk factor.

Ogawa *et al.* ^7^ reported that a large maximum diameter of the portal venous system was a risk factor for PVT. In this study, the diameter of the portal venous system was slightly larger in the PVT group than in the non-PVT group, but there was no significant difference between the two groups. There were some differences between our study and Satoyuki Ogawa’s study. In our study, patients with PVT before PSE were excluded. Satoyuki Ogawa did not exclude patients with PVT before PSE; for those patients with PVT before PSE, thrombus growth was assessed. This might explain why the diameter of the portal venous system was not a risk factor for PVT, as reported by Satoyuki Ogawa.

There were some limitations of the present study. First, this was a single-centre retrospective study, and the number of patients with PVT after PSE was small. A multicenter prospective study with more patients is needed to validate the results. Second, the duration of colour ultrasound before PSE and assessment of PVT after PSE was not constant. Third, although PVT was a complication of PSE, no patients were treated with anticoagulation therapy before and after PSE, and we did not investigate whether patients benefited from anticoagulation therapy. It would be meaningful to investigate whether patients with a high risk of PVT after PSE need anticoagulation therapy.

In conclusion, portal venous blood flow velocity was a factor associated with PVT after PSE, and patients with higher portal venous blood flow velocity were less likely to have PVT after PSE, whereas patients with lower portal venous blood flow velocity were more likely to have PVT. Portal venous blood flow velocity should be considered before patients undergo PSE.

## Ethical approval

The study was approved by the ethics committee of Ganzhou People’s Hospital. The referene number was TY-ZKY2022-015-01.

## Consent

Written informed consent was obtained from the patients for publication and any accompanying images. A copy of the written consent is available for review by the Editor-in-Chief of this journal on request.

## Sources of funding

Not applicable.

## Author contribution

H.L. collected the data, analyzed the relevant information, and drafted the manuscript. J.H. outlined the article and approved the final submission.

## Conflicts of interest disclosure

The authors declare no conflicts of interest for this article. This research did not receive any specific grant from funding agencies in the public, commercial, or not-for-profit sectors.

## Research registration unique identifying number (UIN)

Trial registration number ChiCTR2100048291, It was registered at Chinese Clinical Trial Registry. https://www.chictr.org.cn/bin/userProject.

## Guarantor

Jiaming Huang.

## Data availability statement

The datasets generated and/or analyzed during the current study are available in the Jiaming Huang repository.

## Provenance and peer review

None.
